# Challenges Regarding Protein Provision for the Growing Global Population: Improving the Environmental Impact of Traditional Protein Supply Chains and Maximising use of Coproducts and Alternative, New Resources

**DOI:** 10.1002/gch2.202300059

**Published:** 2023-05-17

**Authors:** Maria Hayes

**Affiliations:** ^1^ Food BioSciences Department Teagasc Food Research Centre Ashtown Dublin 15 Ireland

As the global population continues to grow, overall demand for food will increase by at least 60% by 2030 according to the FAO. Demand for more resource‐intensive foods like meat and dairy products is projected to rise even faster, by nearly 70%. Yet even today, more than 800 million people are malnourished. In addition to this, climate change resulting from greenhouse gas emissions (GHGs) is the challenge of our generation and production of traditionally produced agricultural proteins like meat, dairy, eggs and soy generate emissions, use up terrestrial land mass and use masses of water during their production. Enteric methane is a by‐product of fermentation of feed in ruminants, accounting for approximately 11–14% of agricultural GHGs globally, second only to rice production in terms of agricultural CH_4_ emissions production. However, meat and dairy also make key contributions to human nutrition, food security and livelihoods. Livestock‐derived foods provide essential nutrients that are often lacking in vegan or vegetarian diets, even among those populations with higher incomes. Use of marine ingredients could provide a solution to CH_4_ emissions produced during livestock production, specifically ruminant production. This topic is discussed in article number 2200145, “Potential of Seaweeds to Mitigate Production of Greenhouse Gases during Production of Ruminant Proteins”. This paper details what bioactive compounds in seaweeds can impact positively on the rumen of animals and enhance productivity whilst reducing CH_4_ emissions. The benefits and caveats of the red seaweed *Asparagopsis taxiformis* ‐ considered the gold standard for reduction of CH_4_ from ruminants ‐ are discussed along with the active component bromoform. However, the review paper focuses largely on the potential of other bioactives, found in the brown, red and green seaweeds that show promise as ingredients that may be used as feed additives for CH_4_ mitigation. It also highlights the other animal health benefits associated with these bioactives. For example, the use of polyphenols and phlorotannins specifically is discussed and the potential of these bioactives as CH_4_ reducing ingredients and their use as anthelmintic agents could also potentially reduce the need for antibiotics. Caveats including the cost of seaweeds, the need for controlled production, variable results obtained from different animal trials, the stability of actives when used in different animal feed formulations and processing costs are described.

The marine environment already feeds a third of the world's population. Capture fisheries have played a significant role in this but overfishing has resulted in depletion of marine species and populations. However, article number 2200098 “Maximizing Use of Pelagic Capture Fisheries for Global Protein Supply: Potential and Caveats Associated with Fish and Co‐Product Conversion into Value‐Add Ingredients” highlights how ocean conservation measures in combination with total utilization of fisheries landings is an additional strategy that may contribute significantly to providing the global population with essential, required nutrients and additional health benefits beyond basic, human nutrition with minimum production of greenhouse gases (GHGs) and food waste. Several studies are collated in this article and the review highlights that less than 1% of total global GHGs result from capture fishery activities currently. The benefits and caveats of fish derived proteins are also discussed along with details of how fish protein hydrolysates can be made, the nutritional benefits in terms of fish protein hydrolysate protein digestibility corrected amino acid scores (PDCAAS) and digestible indispensable amino acid score (DIAAS) values, and the health benefits of fish hydrolysates that are sold today, derived from fish species where the bioactives in question are significantly characterised using clinical manufacturing controls and other criteria. It is also highlighted that the blockages concerning growth of pelagic fish processing include a need for investment by Governments and companies in processing equipment that will help current day pelagic fish processors expand their primary processing activities (fileting and packaging) to including enzyme hydrolysis production, filtration, drying and other stabilisation technologies. In addition, there is a need to educate workers and consumers regarding hydrolysate production and the benefits of hydrolysates for health and wellness. The potential markets for fish by‐products/co‐products generated from primary pelagic fisheries processing are highlighted. Potential lucrative markets include functional feed ingredients for the companion animal (pet) treat/functional feeds sector as well as the human functional foods market. Key health targets within these sectors include prevention of inflammation, type 2 diabetes (T2D), maintenance of heart health, prevention of obesity, mental health maintenance and a myriad of other nutritional benefits that can be derived from bioactive ingredients. The paper highlights also the need to ensure compliance with European and other legislation that are in place to protect consumers.

The potential of underutilised fish species as a source of protein is discussed in article number 2200078 “Observations from the Hydrolysis of the Green Sea Urchin (*Strongylocentrotus droebachiensis*)”. The paper describes the biochemical parameters of *S. droebachiensis* and hydrolysates generated from the same as well as the sediment, and shells that result from the hydrolysate process. The amino acid composition and molecular weight distribution of the hydrolysate, and the fatty acid and lipid compositions of *S. droebachiensis* are determined. Additionally, two enzymes and the doses are discussed and the study highlights the commercial use of sea urchins. They are considered an invasive species that threaten local marine species, especially macroalgae, and it is necessary to remove them from the environment. Their use for generation of protein rich hydrolysates is a potentially lucrative method to do so and to create a new market for the non‐gonad fraction of the recovered sea urchins. The authors suggest that the hydrolysates generated which had up to 47.5 % protein (on a dry weight basis) have potential for use in sports nutrition.

The papers listed in this special issue concern improving protein production from traditional sources while reducing the impact of GHG emissions as well as co‐product and invasive species utilisation. Novel alternative proteins are discussed including macroalgae. In article number 2200162 “Big things small packages: An update on microalgae as sustainable sources of nutraceutical peptides for promoting cardiovascular health” highlights peptides generated through microalgal protein hydrolysis that reduce hypertension (by inhibiting angiotensin converting enzyme and endothelial nitric oxide synthase), modulate dyslipidaemia and have antioxidant and anti‐inflammatory properties. The authors suggest that future R&D investments in nutraceutical peptides from microalgae proteins need to focus on the challenges of large‐scale biomass production, improvement in techniques for protein extraction, peptide release and processing, and the need for clinical trials to validate the claimed health benefits as well as formulation of various consumer products. Article number 2200177, “Microalgae‐ sustainable source for alternative proteins and functional ingredients promoting gut and liver health” further highlights how microalgal proteins can impact positively on health, specifically gut and liver health whilst also providing nutritional benefits to consumers. This novel issue highlights the important role marine proteins can play in our diets, societies, and economies now and in the future.

